# Calcium Ionophore-Induced Extracellular Vesicles Mediate Cytoprotection against Simulated Ischemia/Reperfusion Injury in Cardiomyocyte-Derived Cell Lines by Inducing Heme Oxygenase 1

**DOI:** 10.3390/ijms21207687

**Published:** 2020-10-16

**Authors:** Peter Pečan, Szabolcs Hambalkó, Van Thai Ha, Csilla T. Nagy, Csilla Pelyhe, Duško Lainšček, Bence Kenyeres, Gábor B. Brenner, Anikó Görbe, Ágnes Kittel, Monika Barteková, Péter Ferdinandy, Mateja Manček-Keber, Zoltán Giricz

**Affiliations:** 1 National Institute of Chemistry, SI-1000 Ljubljana, Slovenia; peter.pecan@ki.si (P.P.); vanthai.ha@ki.si (V.T.H.); dusko.lainscek@ki.si (D.L.); 2 Graduate School of Biomedicine, Faculty of Medicine, University of Ljubljana, SI-1000 Ljubljana, Slovenia; 3 Department of Pharmacology and Pharmacotherapy, Semmelweis University, 1085 Budapest, Hungary; hambalko.szabolcs@med.semmelweis-univ.hu (S.H.); nagy.csilla@med.semmelweis-univ.hu (C.T.N.); pelyhe.csilla@med.semmelweis-univ.hu (C.P.); bencekenyeres@gmail.com (B.K.); brenner.gabor@med.semmelweis-univ.hu (G.B.B.); gorbe.aniko@med.semmelweis-univ.hu (A.G.); peter.ferdinandy@pharmahungary.com (P.F.); 4 Centre of Excelence EN-FIST, SI-1000 Ljubljana, Slovenia; 5 Pharmahungary Group, 6722 Szeged, Hungary; 6 Institute of Experimental Medicine, ELRN, 1083 Budapest, Hungary; kittel.agnes@koki.hu; 7 Centre of Experimental Medicine, Institute for Heart Research, Slovak Academy of Sciences, 84104 Bratislava, Slovakia; monika.bartekova@savba.sk; 8 Institute of Physiology, Faculty of Medicine, Comenius University in Bratislava, 81372 Bratislava, Slovakia

**Keywords:** extracellular vesicles, TLR4, HO-1, cardioprotection, ischemia/reperfusion injury

## Abstract

Cardioprotection against ischemia/reperfusion injury is still an unmet clinical need. The transient activation of Toll-like receptors (TLRs) has been implicated in cardioprotection, which may be achieved by treatment with blood-derived extracellular vesicles (EVs). However, since the isolation of EVs from blood takes considerable effort, the aim of our study was to establish a cellular model from which cardioprotective EVs can be isolated in a well-reproducible manner. EV release was induced in HEK293 cells with calcium ionophore A23187. EVs were characterized and cytoprotection was assessed in H9c2 and AC16 cell lines. Cardioprotection afforded by EVs and its mechanism were investigated after 16 h simulated ischemia and 2 h reperfusion. The induction of HEK293 cells by calcium ionophore resulted in the release of heterogenous populations of EVs. In H9c2 and AC16 cells, stressEVs induced the downstream signaling of TLR4 and heme oxygenase 1 (HO-1) expression in H9c2 cells. StressEVs decreased necrosis due to simulated ischemia/reperfusion injury in H9c2 and AC16 cells, which was independent of TLR4 induction, but not that of HO-1. Calcium ionophore-induced EVs exert cytoprotection by inducing HO-1 in a TLR4-independent manner.

## 1. Introduction

In ischemic heart disease, one of the leading causes of death—ischemia/reperfusion (I/R) injury—induces different cell death programs, e.g., necrotic and apoptotic pathways (as reviewed in [1]). Despite the intensive research activity in the field over the last 40 years, there is an unmet clinical need for efficacious cardioprotective treatments or interventions [2,3]. A number of mediators and mechanisms have been proposed to be involved in experimental cardioprotective mechanisms, for example, the activation of protein kinase C (PKC), nuclear factor kappa-B (NF-κB), or nitric oxide (NO) pathways (reviewed in [4,5]). In addition, several mediators that can promote cytoprotective responses in the heart, such as heat shock proteins (HSPs) as representatives of damage-associated molecular patterns (DAMPs) [6], adenosine [7], or tumor necrosis factor alpha (TNFα), have been identified [8]. However, to date, no pharmacological tool has reached clinical application for inducing cardioprotection [9]. Therefore, the development of novel cardioprotective treatments is of primary importance. 

Extracellular vesicles (EVs) are membrane-bound particles released by most mammalian cells via diverse mechanisms. They contain a vast array of signaling molecules, rendering EVs prime vehicles of intercellular communication in physiological and pathological processes [10,11]. The use of EVs as experimental therapeutic agents is emerging [12,13,14,15]. Our study was the first to show that EVs were involved in cardioprotection afforded by remote ischemic conditioning (RIC) [16], and studying their use as cardioprotective agents has become popular in recent years [14,17]. Although several mechanisms have been proposed in EV-initiated cardioprotection, to date, none of them have been proven to be sufficiently potent or reproducible to initiate clinical studies with EV-based therapeutic options.

Oxidized phospholipids (oxPLs) are formed in cardiomyocytes after I/R as a result of oxidative burst [18] and can act as DAMPs. We have previously shown that oxPLs generated by oxidative stress can be released from the cells in EVs which induce innate immune responses [19,20]. Although oxPLs may have detrimental effects on cardiomyocytes [18], they have been shown to induce cytoprotective effects in cells against subsequent oxidative stress situations when added in low concentrations [21]. This adaptive response is mediated by the NF-E2-related factor 2/Kelch-like ECH-associated protein 1 (Nrf2/Keap-1) pathway and involves the expression of antioxidant proteins, such as heme oxygenase-1 (HO-1) and thioredoxin reductase 1 (TR1) [22]. A similar response has been observed in macrophages when stimulated with oxPLs and was identified as oxPL-induced macrophage phenotype Mox [23].

Toll-like receptors (TLRs) are evolutionarily conserved proteins present at the membrane surface of immune cells, including dendritic cells, macrophages, natural killer cells, and T and B lymphocytes, but also of non-immune cardiac cells, such as endothelial cells, fibroblasts, and cardiomyocytes [24]. TLRs play a central role in the activation of the innate immune system. These “pattern recognition receptors” (PRRs) recognize microbial molecules termed “pathogen-associated molecular patterns” (PAMPs) and DAMPs, such as oxPLs. Therefore, TLRs discriminate between “self” and “non-self” and activate the innate immune inflammatory response [25]. TLRs, mainly TLR2 and TLR4, have been shown to be associated with cardiovascular disease, as well as with cardioprotection afforded by different interventions [26,27]. It is well-documented that inflammation via TLR-mediated MyD88-dependent NF-κB activation plays an important cytoprotective role in myocardial I/R injury [28], whereas TLR4 is also associated with cardioprotection by the activation of PI3K/Akt signaling, suggesting that there is a crosstalk between TLR and PI3K/Akt pathways in myocardial I/R injury and cardioprotection [29]. Furthermore, the activation or direct supplementation of a major downstream mediator of TLR4 with the tumor necrosis factor alpha (TNFα) was shown to be cardioprotective, even in a large animal model of cardiac I/R injury [8,30,31], which indicates that TLR signaling may be a viable target for novel cardioprotective interventions.

The activation of TLR signaling has also been associated with cardioprotection mediated by EVs. Vicencio et al. documented that the EV-rich fraction isolated from rat and human blood was cardioprotective in different models of cardiac I/R injury [32]. This protection was prevented by the inhibition of HSP70 and by blocking TLR4 in cardiomyocytes, indicating that EVs deliver endogenous protective signals to the heart via a pathway involving TLR4 and cardioprotective HSPs [32]. Very recently, it has also been documented that exosomes derived from miR-146a-modified adipose-derived stem cells decreased the infarct size and reversed myocardial infarction-induced TLR4/NF-κB activation in rats [33]. Since EVs have been shown to be involved in RIC [16], it seems that TLR signaling might mediate EV-induced cardioprotection. However, to utilize TLR-modulating EVs as therapeutic tools, EVs and methods of their production have to meet certain criteria in terms of safety, efficacy, and reproducibility.

To date, TLR4-inducing EVs have only been isolated from blood. In spite of the rapid evolution of EV isolation technologies, EVs obtained from blood have been proven to contain various impurities, and methods are still cumbersome and have moderate yields at best [34,35,36]. In contrast, EVs isolated by more robust methods, such as ultracentrifugation from cell lines that are cultured in well-regulated conditions in chemically defined media, may suffice for the expectations of therapeutic systems. On top of culturing parameters, triggers for EV release can be more standardized in cell cultures than in in vivo interventions. We have previously shown that calcium ionophore A23187, which induces oxidative stress, stimulates the shedding of EVs from human embryonal kidney 293 (HEK 293) cells that activate TLR4 via oxPLs [19,20], indicating that non-microbial signals may also be sufficient for triggering cardioprotection via TLR4 induction.

The aims of this study were to decipher whether stressEVs released after A23187 calcium ionophore treatment, which has the ability to induce the release of TLR4-activating EVs from cell cultures, can induce signaling in H9c2 and AC16 heart-derived proliferating cell lines and to assess if such EVs are capable of inducing cardioprotection against acute I/R injury. We showed that stressEVs induced TLR4-dependent signaling in macrophages and H9c2 and AC16 cells, but only induced adaptive signaling in heart-derived cells. Furthermore, adaptive signaling induced by stressEVs contributed to cardioprotection via HO-1 activity in a TLR4-independent manner.

## 2. Results

### 2.1. Calcium-Ionophore Treatment Induces the Release of StressEVs from HEK293 Cells

**Figure 1:**
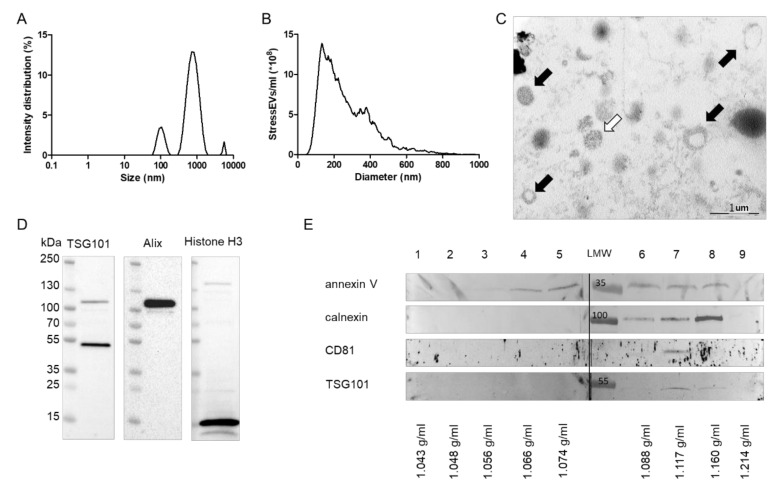
Characterization of stressextracellular vesicles (EVs). HEK293 cells were treated with calcium ionophore for 1 h and stressEVs were isolated. (**A**) Dynamic Light Scattering (DLS) analysis of an isolate showing the intensity distribution of EVs. (**B**) Nanoparticle tracking analysis (NTA) of EV preparations. (**C**) Electron microscopic images of a representative EV preparation. Black arrows: EVs, and white arrow: nuclear material. (**D**,**E**) Western blot analysis of EVs showing that EVs and nuclear material were present in our preparations.

Dynamic light scattering (DLS) analysis revealed that particles (referred to as stressEVs) that were released after HEK293 cells were treated with A23187 included large EVs (100–1000 nm in diameter) and a population with a diameter smaller than 100 nm (small EVs). A minor subpopulation with a diameter greater than 1000 nm was also present in the stressEV preparations (Figure 1A). Nanoparticle tracking analysis (NTA) also confirmed that stressEV preparations were dominated by EVs with an average size of 250 nm (Figure 1B). Electron microscopic imaging confirmed the presence of EVs in our stressEV preparations (Figure 1C, black arrows). The presence of presumably nuclear material was also evident in certain EVs (Figure 1C, white arrow).

To further describe the contents of our stressEV preparations, we performed Western blot analysis against Alix and TSG101, which are well-accepted markers of EVs. By detecting histone H3 in EV preparations, we also confirmed the presence of nuclear material (Figure 1D). However, the absence of the cleaved caspase 3 signal proved that our EV preparations did not contain apoptotic bodies). StressEVs were then separated by density gradient ultracentrifugation, which revealed that EV markers CD81 (in Fraction 7), TSG101 (in Fraction 7 and 8), and calnexin (in Fraction 6–8), as well as membrane marker annexin V (in Fraction 3–8), appeared at the density range characteristic of EVs (Figure 1E). Differences in the marker distribution also implied the heterogeneity of stressEVs.

### 2.2. StressEVs Activate the TLR4 Receptor, but Not the Adaptive Response in Macrophages

**Figure 2:**
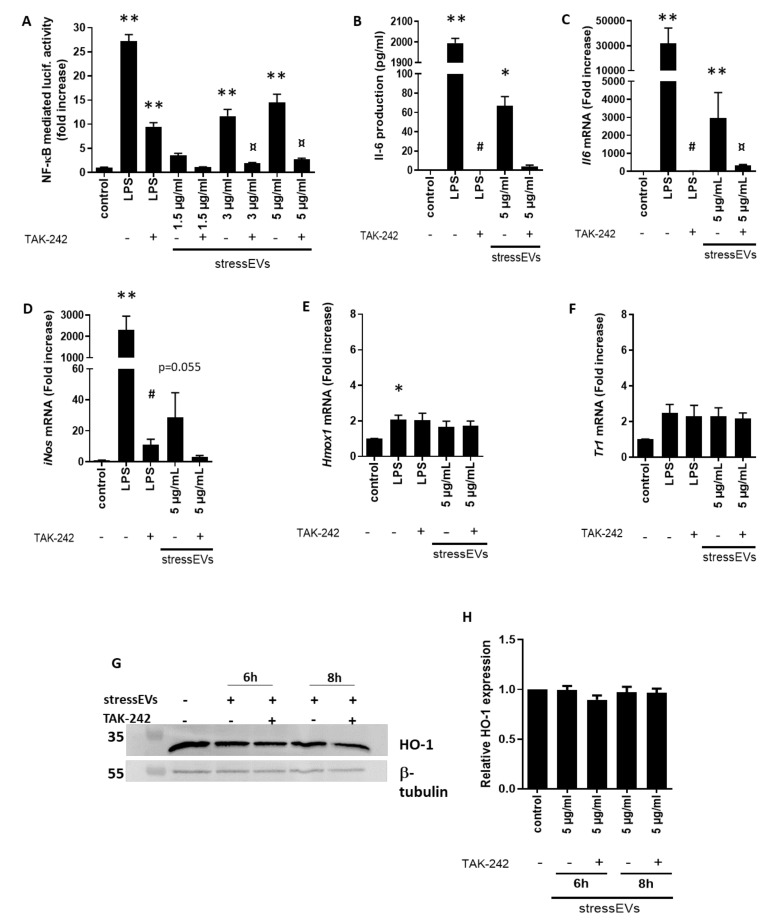
StressEVs activate TLR4, but do not induce an adaptive response in macrophages. (**A**) HEK293T cells were transfected with plasmids for hTLR4, hMD-2/CD14, firefly luciferase under the nuclear factor kappa-B (NF-κB) promoter, and *Renilla* luciferase for normalization. Cells were stimulated with stressEVs (1.5, 3, or 5 µg/mL) or lipopolysaccharide (LPS) (10 ng/mL) for 24 h, with or without 2.5 μM TAK-242. A dual luciferase test was performed. Negative controls were transfected but unstimulated cells. Data were pooled from two independent experiments (*n* = 6; ** *p* < 0.01 vs. control; ¤ *p* < 0.05 vs. stressEVs). (**B**–**F**) Macrophages were stimulated for 6 h (16 h for ELISA) with stressEVs (5 μg/mL) or LPS (10 ng/mL) in the absence or presence of TAK-242 (2.5 μM). *Il6*, *iNos*, *Txnrd1*, and *Hmox1* mRNA levels were determined using qPCR and Il-6 by ELISA, respectively. (**G**,**H**) Macrophages were stimulated for 6 or 8 h with stressEVs (5 μg/mL) in the absence or presence of TAK-242 (2.5 μM). Heme oxygenase 1 (HO-1) expression was detected using WB. β-tubulin expression was used as the loading control. Data were pooled from four (**C**–**F**; *n* = 5) or two (**B**; *n* = 6) independent experiments (* *p* < 0.05 vs. control; ** *p* < 0.01 vs. control; # *p* < 0.05 vs. LPS; ¤ *p* < 0.05 vs. stressEVs). WB is a representative of two independent experiments and optical density plot analysis of band intensities (**H**) was performed from two independent experiments.

StressEVs induced TLR4 receptor signaling, which was confirmed first by NF-κB activation in HEK293T cells expressing the TLR4/MD-2/CD14 receptor complex treated with increasing concentrations of stressEVs in the presence or absence of TLR4 inhibitor TAK-242 (Figure 2A). For the positive control, we used lipopolysaccharide (LPS).

StressEVs were shown to promote inflammation in a TLR4-dependent manner through oxPLs [19,20]. OxPLs were shown to induce a special type of macrophages—a Mox type [23]—determined with the expression of Nrf2-mediated redox-regulatory genes, such as *Hmox1*, with protective effects. Pro-inflammatory *Il6* (Figure 2B,C) and inducible NOS (*iNos*) (Figure 2D) mRNA expression, but not *Hmox1* or *Txnrd1* expression, was detected in mouse macrophages after stressEV stimulation (Figure 2E,F). Similarly, stressEVs did not influence the HO-1 protein expression in macrophages (Figure 2G,H). These data show that stressEVs induce pro-inflammatory cytokines in a TLR4-dependent manner, but are not able to induce a Mox phenotype or adaptive response in macrophages.

### 2.3. StressEVs Activate Cardioprotective Signaling in H9c2 and AC16 Cell Lines

**Figure 3:**
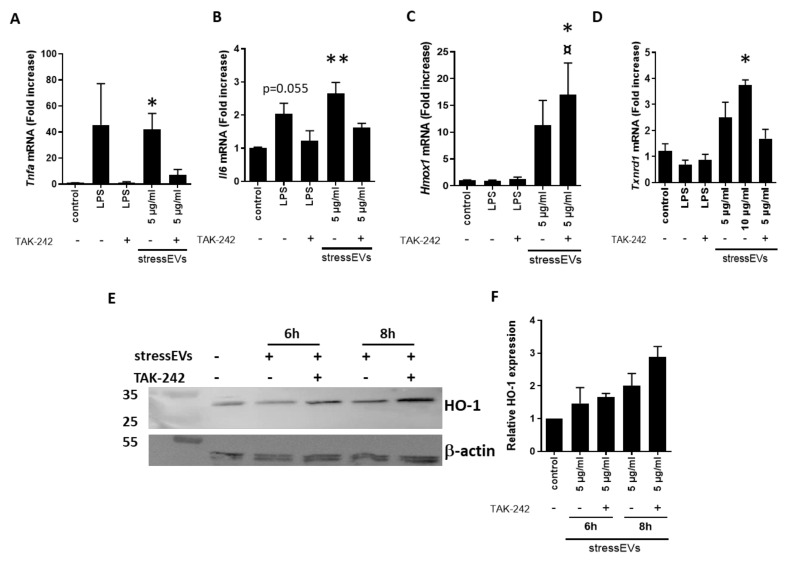
StressEVs activate cardioprotective signaling pathways in H9c2 cells, independently of TLR4 signaling. (**A**–**D**) H9c2 cells were stimulated for 6 h with stressEVs (5 or 10 μg/mL) or LPS (10 ng/mL) in the absence or presence of TAK-242 (2.5 µM). *Tnfa Il6*, *Txnrd1*, and *Hmox1* mRNA levels were determined using qPCR. Data were pooled from four independent experiments (*n* = 5; * *p* < 0.05 vs. control; ** *p* < 0.01 vs. control; ¤ *p* < 0.05 vs. stressEVs). (**E**,**F**) H9c2 cells were stimulated for 6 or 8 h with stressEVs (5 µg/mL) in the absence or presence of TAK-242 (2.5 µM). HO-1 expression was detected using WB. β-actin expression was used as the loading control. WB is a representative of two independent experiments and optical density plot analysis of band intensities (**F**) was performed from two independent experiments.

Furthermore, we investigated the effect of stressEVs on the mRNA expression of genes downstream of TLR4 in H9c2 cells. Both LPS and EVs induced the expression of *Tnfa* and *iNOS* mRNA (Figure 3A and Supplementary Figure S1). Preincubation of the cells using the TLR4 inhibitor TAK-242 decreased *Tnfa* and *iNOS* expression after LPS, as well as stressEV stimulation, confirming TLR4-dependent signaling (Figure 3A and Supplementary Figure S1). Interestingly, the expression of pro-inflammatory *Il6,* which was strongly expressed in macrophages (Figure 2B), was minor, but still TLR4-dependent (Figure 3B). H9c2 cells are poor producers of pro-inflammatory cytokines after TLR4 activation [37,38]. Due to a low mRNA expression, we were unable to detect cytokines in the supernatant. On the other hand, the mRNA expression of cytoprotective genes *Hmox1* (HO-1) and *Txnrd1* (TR1) was elevated in the stressEV-treated groups compared to the control (Figure 3C–F). TLR4 ligand LPS did not induce their expression, suggesting that stressEVs might influence cytoprotective pathways via signaling mechanisms other than TLR4 in H9c2 cells. As expected, TAK-242 inhibited all LPS-induced gene expression in H9c2 cells (Figure 3), but it was not able to inhibit *Hmox1* mRNA and protein expression after stressEV stimulation. Moreover, the expression of HO-1 after stressEV and TAK-242 treatment was even increased on mRNA, as well as the protein level (Figure 3C,E,F).

**Figure 4:**
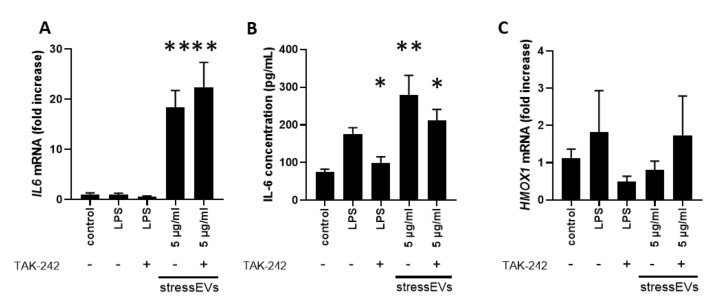
StressEVs activate interleukin signaling in AC16 cells. AC16 cells were stimulated for 6 h with stressEVs (5 µg/mL) or LPS (10 ng/mL) in the absence or presence of TAK-242 (2.5 µM). *IL6* and *HMOX1* mRNA levels were determined by qPCR (**A**,**C**) (*n* = 6; ** *p* < 0.01 vs. control), and IL-6 expression was detected using ELISA (**B**) (*n* = 3; ** *p* < 0.01 vs. control; * *p* < 0.05 vs. control).

We also assessed the key transcriptional effects of stressEVs in AC16 human cardiomyoblast cells. Similar to what was seen in H9c2 cells, we found that *IL6* gene and protein expression was induced by stressEVs and secretion was decreased by TLR4 inhibition (Figure 4A,B). However, in contrast to the data from H9c2 cells, *TNFA* mRNA was below the detection limit, and *HMOX1* expression was not elevated by stressEVs in AC16; only a tendency was observed when stressEVs were applied in combination with the TLR4 inhibitor TAK-242 and when they were treated with the TLR4-inducer LPS (Figure 4C), which indicates that HMOX1 may be under complex regulation by TLR4 in AC16 cells.

### 2.4. StressEVs Protect against Simulated I/R-Induced Cytotoxicity via the Induction of HO-1, Independently of TLR4 Receptor Signaling

As stressEVs induced protective signaling in H9c2 and AC16 cells, we were interested whether they can have cardioprotective effects.

**Figure 5:**
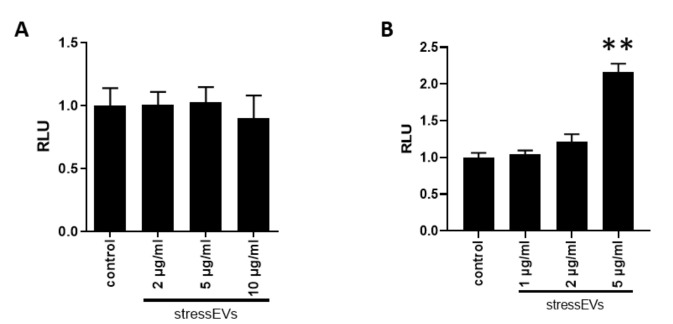
StressEVs do not activate apoptosis or induce cytotoxicity. (**A**,**B**) Caspase 3/7 activity was measured after 4 h incubation of the H9c2 (**A**) or AC16 (**B**) cells with increasing concentrations of stresses. Data were pooled from three (**A**; *n* = 9; **B**; *n* = 6) independent experiments (** *p* < 0.01 vs. control).

We investigated whether stressEVs induce apoptosis in H9c2 and AC16 cells to assess their safety in cardiomyocytes. StressEVs up to a 2 μg/mL concentration did not induce apoptosis in any of the cell lines. In AC16 cells, a 5 μg/mL concentration triggered caspase 3/7 activation; however, stressEV-treated H9c2 cells did not induce apoptosis (Figure 5A,B).

**Figure 6:**
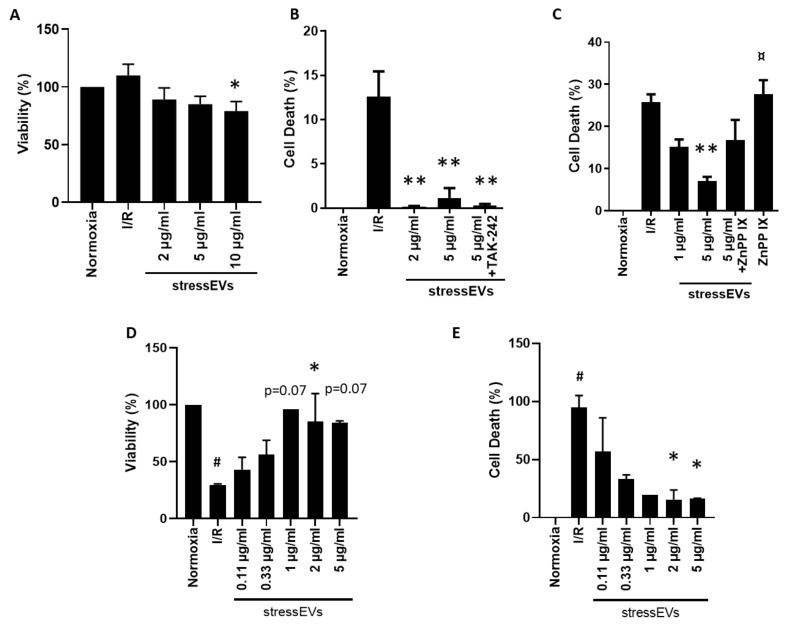
StressEVs protect against simulated I/R-induced cytotoxicity via HO-1 activity. (**A**) H9c2 cells were incubated for 4 h with increasing concentrations of stressEVs in normoxic conditions. The cell viability of H9c2 cells was determined by the Calcein-AM assay. (**B**,**C**) H9c2 cells were pre-incubated with either TAK-242 (2.5 µM) or ZnPP IX (1 µM) for 1 h prior to stressEV stimulation (1 or 2 and 5 µg/mL) for 6 h before I/R. LDH release was determined. (**D**,**E**) AC16 cells were preincubated with stressEVs (0.1–5 µg/mL) for 4 h before I/R. Viability was measured by the Calcein-AM assay (**D**) and cytotoxicity was determined by the CytoTox-Glo assay (**E**). Data are representative of three (**A**–**C**; *n* = 5) or two (**D**–**E**; *n* = 6) independent experiments (* *p* < 0.05 ** *p* < 0.01 vs. I/R; # *p* < 0.05 vs. normoxia; ¤ *p* < 0.05 vs. 5 µg/mL stressEVs + ZnPP IX).

We also evaluated the toxicity of stressEVs. As shown in Figure 6A, low concentrations of EVs have no negative effect on the viability of H9c2 cells in normoxic conditions. The highest examined concentration of stressEVs (10 μg/mL) decreased the viability of H9c2 cells moderately, similar to their apoptotic effects (Figure 5A). Therefore, a maximum of 5 μg/mL was used for further experiments. We investigated the potential cytoprotective effects of a pretreatment with stressEVs on rodent H9c2 (Figure 6B) or human AC16 (Figure 6E) cells subjected to simulated I/R. The cytoprotective effect of stressEVs was observed by measuring the release of LDH from H9c2 cells (Figure 6B) or by measuring the viability (Figure 6D) or cell death (Figure 6D) in AC16 cells after 16 h of ischemia under 1% O_2_ in hypoxic solution and 2 h of reoxygenation in media. Therefore, stressEVs released after calcium ionophore treatment showed a protective effect against I/R in heart-derived human and rodent cell lines.

To decipher the molecular mechanism responsible for the cytoprotective effect of stressEVs, we decided to assess whether it could be abolished by the inhibition of TLR4 signaling. We observed that the inhibition of TLR4 signaling by TAK-242 did not abolish cytoprotection by EVs in H9c2 cells (Figure 6B), which suggests that our EV preparations induced their cytoprotective effects via signaling pathways other than TLR4. In adaptive response situations induced by lipid peroxidation products, cytoprotection is mediated through the Nrf2 pathway, where HO-1 is mainly expressed [22]. The role of increased HO-1 expression in cardioprotection has also already been described [39]. As we observed an increased expression of HO-1, we were interested whether stressEVs mediate cytoprotection through HO-1. Indeed, cytoprotection by stressEVs was reduced by the inhibition of HO-1 by the co-treatment of H9c2 cells with HO-1 inhibitor zinc protoporphyrine IX (ZnPP IX) (Figure 6C). These results indicate that the cytoprotective effect of EVs is mediated through HO-1 via mechanisms independent of TLR4 signaling in H9c2 cells. Similar results were obtained in AC16 human cells, showing that 2 to 5 μg/mL stressEVs decreased sI/R-induced cell death (Figure 6D,E).

## 3. Discussion

Here, we present evidence that a short time exposure of HEK293 cells to calcium ionophore A23187 triggers stressEV release, which induces TLR4 signaling and pro-survival mechanisms in H9c2 and AC16 cells, but not in macrophages. Furthermore, we provide evidence that the cytoprotection afforded by calcium ionophore-induced stressEVs is mediated by HO-1, independently of their effect on TLR signaling.

The characterization of stressEVs revealed several EV markers. Interestingly, there was a strong presence of annexin V in almost all fractions after gradient ultracentrifugation and a minor presence of calnexin in higher density fractions, indicating that stressEV preparations consist of several types of EVs. The presence of annexin V may be related to the formation of stressEVs, since Gong et al. showed that MLKL-dependent Ca-influx promoted phosphatidylserine exposure and that ESCRT-III facilitates the shedding of damaged annexin V plasma membrane bubbles, thus contributing to cell survival [40].

As stressEVs were shown to activate TLR4 through oxPLs [19,20], we were interested whether they can induce cardioprotection. StressEVs are released after applying oxidative stress to the cells. Oxidative stress damages all biologically important molecules and some act as DAMPs. Our results on the induction of TLR4 confirm previous findings that EVs can induce an immune response [6,32], but on the contrary, TLR4-dependent activation was not cardioprotective. H9c2 and AC16 cells seem to express low amounts of TLR4 or poorly express pro-inflammatory cytokines. Although the mRNA expression of *Tnfa* was evident and TLR4-dependent, we and also others [37] were not able to detect cytokine secretion from the H9c2 cell line, or it was low when a very high amount of LPS (10 µg/mL) was used [38], but intracellular pathways were activated. As the direct supplementation of TNFα can achieve cardioprotection [8], this might be the reason we were unable to observe it and it also implies the indirect role of TLR4 signaling in cardioprotection. In addition, although Davidson et al. and Vicencio et al. showed that rat and human EVs isolated from blood induced cardioprotection through HSP70 and TLR4 signaling [6,32], Davidson also showed that EVs isolated from HUVEC cells protected cardiomyocytes from I/R, although no HSP70 was detected. These observations and ours here, that stressEVs protect AC16 cells, even in the absence of the induction of HMOX1, show that yet to be identified signaling pathways are also involved in cardioprotection by EVs.

EVs are very complex entities, where various molecules may influence an unknown number of signaling pathways. This might be a drawback of EV therapies, as we also showed that stressEVs may only have a narrow therapeutic window, since higher doses of stressEVs decreased the viability of H9c2 cells and induced apoptosis, even in normoxic conditions. Nevertheless, since, in this study, EV release was triggered under well-controlled, chemically-defined conditions, the obtained stressEVs might show a safety profile that may afford translation of the current results, which is substantiated by the finding that cardioprotection was achieved in heart-derived proliferating cells of rat and human origin, despite the induction of pro-inflammatory signaling pathways.

Here, we found that the induction of an antioxidant response via the upregulation of HO-1 and TR1 expression may be a direct mechanism of the cardioprotective effects of stressEVs in H9c2 and AC16 and that TLR4 induction might even be dispensable for cytoprotection. HO-1 induction was previously found to convey cytoprotective effects against various harmful stimuli, including high glucose- or cobalt-induced chemical stress in cardiomyocytes [41] and hypoxia-induced injury in H9c2 cells [42,43], and against coronary occlusion-induced I/R injury in vivo [44]. However, the latter study found that HO-1 induction reduced plasma TNFα levels [44], which is in contrast to our finding. The induction of antioxidant systems, in particular the HO-1, was achieved via the application of EVs. In a study by Mahmoud et al., TNFα-stimulated endothelial cell-derived EVs protected recipient cells against oxidative stress [45], but their effects on cardiomyocytes were not assessed. Tr1 plays a role in restoring oxidized proteins via thioredoxin, which alleviates the effects of myocardial infarction [46]. These findings demonstrate that oxidative stress, including cardiac I/R injury, can be alleviated by stressEVs via the induction of pro-survival programs.

As stressEVs were unable to induce HO-1 or TR1 in macrophages, we concluded that their contribution to the cardioprotection induced by stressEVs may not be significant. On the other hand, the results from I/R experiments on H9c2 cells imply that stressEV-mediated protection was due to an increased HO-1 expression in H9c2 and AC16 cells and that the inhibition of pro-inflammatory signaling might contribute to higher protection. This is in agreement with some previous observations, where the innate immune system in the acute response to I/R contributed to cell death and myocardial infarction (reviewed in [47]).

In summary, here, we demonstrated, for the first time, that EVs released from cells cultured in vitro as a consequence of calcium ionophore treatment can be utilized as a treatment option against simulated I/R injury in cardiac myocytes and that the key mechanism of this cytoprotective signal is the induction of antioxidant pathways such as HO-1 and TR1. Based on these results, we suggest that calcium ionophore-induced stressEVs may reveal novel avenues for cardioprotective treatments against ischemic cardiac disease such as myocardial infarction.

## 4. Materials and Methods

### 4.1. Reagents

All reagents were purchased from Merck (Darmstadt, Germany), unless otherwise noted. LPS (from Salmonella abortus equi HL83) was received from K. Brandenburg (Forschungszentrum Borstel, Germany). TAK-242 (CLI-095) was purchased from InvivoGen (San Diego, CA, USA).

### 4.2. Cell Culturing

HEK293 and HEK293T cells were grown in DMEM containing 10% heat-inactivated fetal bovine serum (FBS) (both Invitrogen Life Technologies, Carlsbad, CA, USA). Mouse macrophages (immortalized BMDMs) were grown in RPMI 1640 containing 10% heat-inactivated FBS (both Invitrogen Life Technologies). H9c2 rat cardiomyoblast cells and AC16 human cardiac cells were obtained from ECACC and cultured in high glucose (4500 mg/L) Dulbecco’s modified Eagle’s medium (DMEM) supplemented with 10% FBS, 2 mM glutamine, and 100 U/mL of penicillin and streptomycin.

### 4.3. Isolation and Characterization of StressEVs

HEK293 cells were seeded at a 1.5 × 10^6^ cells/mL density in DMEM containing 10% heat-inactivated FBS (Invitrogen). The next day, growth media were removed and cells were washed with HBSS. Next, we stimulated the cells with 10 μM calcium ionophore A23187 in HBSS buffer (Invitrogen) containing 2.5 mM CaCl_2_ without FBS. StressEVs were collected after 1 h stimulation of HEK293 cells. To remove cells, supernatants were centrifuged at 800× *g*. StressEVs were isolated by ultracentrifugation at 100,000× *g* (rotor type 50TI; Beckman Coulter) two times. StressEVs were resuspended in PBS and their concentration was determined by the BCA assay.

To further analyze stressEVs, gradient ultracentrifugation was performed. OptiPrep (60 *w*/*v*% iodixanol was diluted to 40%, 20%, 10%, and 5% in 0.25 M sucrose in 10 mM Tris-HCl, pH 7.5) and a discontinuous gradient were formed by layering 2.5 mL of each solution in 10 mL polypropylene centrifugation tubes (Beckman Coulter, Miami, FL, USA). A total of 250 µL (120 μg) of stressEVs was layered on the top. The samples were centrifuged at 100,000× *g* (rotor type 50TI, Beckman Coulter) for 16 h at 4 °C. Then, 1 mL fractions of density gradient layers were collected from the top (Fraction 1–Fraction 9). The density of fractions was calculated based on the iodixanol concentration measured by spectrophotometry at 340 nm on SynergyMx (BioTek, Winooski, VT, USA).

The hydrodynamic average particle size of stressEVs was measured by the Zetasizer Nano (Malvern Instruments, Malvern Hills, UK) Dynamic Light Scattering (DLS) apparatus at 20 °C, using an angle of 173o and a 633-nm laser.

The size distribution of stressEVs was assessed by nanoparticle tracking analysis by the Nanosight NS300 instrument (488 nm laser) connected to a sample assistant for automated sample processing (both Malvern Panalytical, UK) with a constant syringe pump flow. Five recordings of 60 s were performed and captured at camera level 13. After a visual inspection of all records, the raw data were analysed using the NanoSight NTA 3.3 software (Malvern Panalytical, Malvern, UK). Automatic settings were selected for the minimum expected particle size and blur, the minimum track length was set to 10, the detection threshold to 5, the sample viscosity to the corresponding viscosity for water, and the temperature to 25 °C. The output data were calculated as the EV size (mean and modal hydrodynamic diameter in nm).

EVs were visualized by transmission electron microscopy. Vesicle pellets were fixed with 4% formaldehyde, postfixed in 1% OsO_4_. EVs were block-stained with 1% uranyl acetate in 50% ethanol, and then dehydrated in graded ethanol and embedded in Taab 812 (Taab Laboratories, Aldermaston, UK). Ultrathin sections were cut and analyzed with a Hitachi 7100 electron microscope.

For Western blotting, H9c2 cells were seeded at 1 × 10^6^ cells/well in a 6-well plate and were preincubated with TAK-242, where indicated, for 1 h prior to stressEV (5 µg/mL) stimulation for 6 or 8 h. Cells were washed with PBS and lysed in 100 μL. Cell or stressEV samples were lysed in radioimmunoprecipitation assay buffer (Cell Signaling Technology, Danvers, MA, USA) supplemented with 1 mM PMSF (Roche, Basel, Switzerland), 0.1 mM sodium fluoride, 200 mM sodium orthovanadate, and complete protease inhibitor cocktail (Roche). SDS-PAGE and WB were performed. Primary antibodies used were anti-TSG101 (Abcam, Cambridge, UK ab83), anti-Alix, anti-histone H3 and anti-cleaved caspase 3 (Cell Signaling Technology #2171, #4499 and #9664), anti-annexin V (ab108194), anti-calnexin (ab22595), anti-heme oxygenase 1 (ab13243; Abcam), and anti-CD81 (sc-166029, Santa Cruz Biotechnology, Dallas, TX, USA). Horseradish peroxidase-conjugated secondary antibodies were used to detect the proteins.

### 4.4. Assessment of TLR4 Induction and the Activation of Cardioprotective Signaling

HEK293T cells (2 × 10^4^ cells/well) were transfected with expression plasmids for pFlag-CMV-hTLR4 (a gift from Carsten Kirschning, University of Duisburg-Essen,Germany), pDUO-hMD-2/hCD14 (Invivogen), pELAM1-luciferase (NF-κB promotor) (a gift from Carsten Kirschning), and phRL-TK Renilla luciferase (Promega, Madison, WI, USA) using PEI transfection reagent (12 μL/1 μg DNA). Cells were stimulated with stressEVs or LPS as a positive control for 24 h. Cells were lysed and analyzed for luciferase activity using a dual luciferase assay on the Orion luminometer (Berthold).

Where indicated, cells were preincubated with TAK-242 (2.5 μM) for 1 h prior to stressEVs or LPS (as a positive control) for 6 h. Total RNA was isolated using TRIZOL reagent (Roche). cDNA was prepared using a high capacity cDNA reverse transcription kit (Applied Biosystems, Foster City, CA, USA) and qPCR for *Il6*, *iNos*, *Hmox1*, *Tnfa*, *Il6*, *Hmox1*, and *Txnrd1* was performed using the SYBR green I master kit (Roche) on LightCycler 480 (Roche). The results are displayed as the cycle of quantification (Cq) expression relative to *Gapdh* and presented as the fold increase relative to untreated cells (primer sequences are given in Table 1).

### 4.5. Assessment of Toxicity and Cardioprotection by EVs

H9c2 cells were seeded in 96-well plates at 10^4^/well in normal growth medium. After 24 h, growth media were replaced with growth media containing a vehicle or EV in increasing doses and the cells were kept in a CO_2_ incubator (Scancell-Labogene, Lynge, Denmark) for 4 h. Then, in groups of cells, growth media were replaced for 16 h with either normoxic solution (in mM: NaCl 125, KCl 5.4, NaH_2_PO_4_ 1.2, MgCl_2_ 0.5, HEPES 20, MgSO_4_, 1.3, CaCl_2_ 1, glucose 15, taurine 5, creatine-monohydrate 2.5 and BSA 0.1%, pH 7.4) in a CO_2_ incubator (Norm groups) or hypoxic solution (in mM: NaCl 119, KCl 5.4, MgSO_4_ 1.3, NaH_2_PO_4_ 1.2, HEPES 5, MgCl_2_ 0.5, CaCl_2_ 0.9, Na-lactate 20, BSA 0.1% pH 6.4; I/R groups) in a three-gas (95% N_2_ and 5% CO_2_, with a maximum of 1% O_2_) incubator (Panasonic Healthcare Co., Ltd., Gunma, Japan), both containing the above-mentioned doses of EVs or vehicle only [48]. Following the 16 h normoxic or simulated I/R conditions, cells were kept in growth medium in an incubator supplied with 95% air and 5% CO_2_.

To assess the cell viability, calcein staining was performed as described previously [49]. Briefly, cells were washed with warm DPBS and calcein solution (1 µM) was added and incubated for 30 min at room temperature in a dark chamber. Then, the calcein solution was replaced with fresh DPBS. An unbiased evaluation was performed by automatic detection of the fluorescence intensity of each well by the Varioskan Lux multimode microplate reader (Thermo Fisher Scientific, Waltham, MA, USA) at a temperature of 37 °C; excitation wavelength of 490 nm, and emission wavelength of: 520 nm. Cell survival results are shown as relative fluorescence units (RFU) normalized to that of the normoxic group (Norm).

Necrotic cell death due to simulated I/R or stressEV treatment was assessed from cell supernatants of H9c2 cardiomyoblasts. Cells were seeded in 96-well plates at 3 × 10^4^/well in DMEM (with 4.5 g/L glucose) containing 10% heat-inactivated FBS. The next day, cells were pretreated with TAK-242 (2.5 μM) or ZnPP IX (1 µM) for 1 h and stimulated with stressEVs (1 or 5 µg/mL) for 6 h under normal O_2_. Then, in groups of cells, growth media were replaced for 16 h with either normoxic solution under normal O_2_ or with hypoxic solution in a three-gas incubator, as above. Following the 16 h normoxic or sI/R conditions, cells were kept in growth medium in an incubator under normal O_2_. For the LDH assay, the supernatant of cells was acquired by centrifugation, for 3 min at 250× *g*, and analyzed for the presence of LDH activity with the CyQUANT LDH Cytotoxicity Assay (Thermo Fisher Scientific). Absorbance at 490 nm was measured using the SynergyMx (BioTek, Winooski, VT, USA) multiplate reader. Supernatant from cells, treated with 1% detergent Triton X-100, was used as a positive control (100% LDH release), and supernatant from untreated cells was used as a negative control.

Cell viability was also assessed in AC16 cells by the CytoTox-Glo cytotoxicity assay (Promega, Madison, WI, USA), according to manufacturers’ instructions.

### 4.6. TNF-α and IL-6 ELISA

To quantify protein expression by ELISA, AC16 cells were seeded at 1 × 10^6^ cells/well in a 6-well plate and preincubated with TAK-242, where indicated, for 1 h prior to stressEV (5 µg/mL) stimulation for 6 h. Cell culture supernatants were collected and analyzed by Human TNF-alpha DuoSet ELISA and Human IL-6 DuoSet ELISA (R&D Systems, Minneapolis, MN, USA), according to manufacturers’ instructions.

Macrophages were seeded in 96-well plates at 1 × 10^5^/well. After 24 h, growth media were removed and, where indicated, cells were preincubated with TAK-242 (2.5 μM) for 1 h prior to the addition of stressEVs or LPS. Supernatants were collected after 16 h and analyzed for IL-6 (Invitrogen).

### 4.7. Apoptosis Assay

For Caspase 3/7 activity measurement, AC16 and H9c2 cells were seeded 1 × 10^4^ cells/well in a white-walled 96-well plate and were stimulated with stressEVs (1, 2, or 5 μg/mL) for 4 h. Cells were analyzed for caspase 3/7 activity using the Caspase-Glo 3/7 Assay System (Promega, Madison, WI, USA), according to manufacturers’ instructions, on a Varioscan LUX multimode microplate reader (Thermo-Fisher, Waltham, MA, USA).

### 4.8. Statistical Analysis

Values are expressed as means ± SEM. Statistical analysis was performed between groups by ANOVA with Tukey’s post-hoc test or by Kruskal–Wallis One-Way Analysis of Variance on Ranks with Dunn’s post-hoc test in the case of datasets with a non-normal distribution (Figure 2B,C,D, Figure 3A,B,E, Figure 5B and Figure 6D) by using GraphPad Prism 8 software. A *p* < 0.05 value was considered significant.

## Figures and Tables

**Table 1:** Primers used for qPCR.

Mouse	Forward 5′→3′	Reverse 5′→3′
Gapdh	TTCACCACCATGGAGAAGGC	GGCATGGACTGTGGTCATGA
Il1beta	AAGGAGAACCAAGCAACGACAAAA	TGGGGAACTCTGCAGACTCAAACT
Hmox1	CAGGATTTGTCTGAGGCCTT	CATAGACTGGGTTCTGCTTGT
Txnrd1	GCTGGTCTTGGATTTTGTCAC	CTTCACTGTGTCTTCGACTTTC
Il6	CGGAGGCTTAATTACACATGTTC	CTGGCTTTGTCTTTCTTGTTATC
iNos	GCCATTGAGTTCATCAACCAGTA	CTGGTAGGTTCCTGTTGTTTCTA
**Rat**	**Forward 5′→3′**	**Reverse 5′→3′**
Gapdh	GTATTGGGCGCCTGGTCACC	CGCTCCTGGAAGATGGTGATGG
Tnfalpha	ACTGAACTTCGGGGTGATTG	GCTTGGTGGTTTGCTACGAC
Hmox1	GATTTGTCCGAGGCCTTGAA	GTTCTGCTTGTTTCGCTCTATC
Il6	TGATGGATGCTTCCAAACTG	GAGCATTGGAAGTTGGGGTA
Txnrd1	GCCAAATTTGACAAGAAGGTGA	CTTTCAGAGCTTGTCCTAACAGA
**Human**	**Forward 5′→3′**	**Reverse 5′→3′**
HPRT	AGATGGTCAAGGTCGCAAG	TTCATTATAGTCAAGGGCATATCC
TNFA	CCTGTGAGGAGGACGAAC	CGAAGTGGTGGTCTTGTTG
HMOX1	GCCCCAGGATTTGTCAGAG	CATAGATGTGGTACAGGGAG
IL6	ACAGCCACTCACCTCTTC	AAGTCTCCTCATTGAATCCAG

## Supplementary Materials

The following are available online at https://www.mdpi.com/1422-0067/21/20/7687/s1.

## Abbreviations

Alix	ALG-2-interacting protein X
CD81	Cluster of differentiation 81
DAMP	Damage-associated molecular pattern
DLS	Dynamic light scattering
DMEM	Dulbecco’s modified Eagle’s medium
DOAJ	Directory of open access journals
DPBS	Dulbecco’s phosphate-buffered saline
ECACC	The European Collection of Authenticated Cell Cultures
ESCRT-III	Endosomal Sorting Complexes Required for Transport-III
EV	Extracellular vesicle
FBS	Fetal bovine serum
HBSS	Hank’s Balanced Salt Solution
HEK293	Human embryonal kidney 293
HO-1	Heme oxygenase 1
HSP	Heat shock protein
IL-6	Interleukin 6
iNOS	Nitric oxide synthase, inducible
I/R	Ischemia/reperfusion
LD	Linear dichroism
LDH	Lactate dehydrogenase
LPS	Lipopolysaccharide
MD-2	Lymphocyte antigen 96
MDPI	Multidisciplinary Digital Publishing Institute
MLKL	Mixed lineage kinase domain-like protein
MyD88	Myeloid differentiation primary response 88
NF-κB	Nuclear factor kappa-B
NO	Nitric oxide
Nrf2/Keap-1	NF-E2-related factor 2/Kelch-like ECH-associated protein 1
NTA	Nanoparticle tracking analysis
oxPL	Oxidized phospholipid
PAMP	Pathogen-associated molecular patterns
PBS	Phosphate buffered saline
PI3K/Akt	Phosphatidylinositol 3-kinase/Akt
PKC	Protein kinase C
PRR	Pattern recognition receptor
RIC	Remote ischemic conditioning
RPMI 1640	Roswell Park Memorial Institute 1640 Medium
TLA	Three letter acronym
TLR	Toll-like receptor
TNFα	Tumor necrosis factor alpha
Tr1	Thioredoxin reductase 1
TSG101	Tumor susceptibility gene 101
ZnPP IX	Zinc protoporphyrine IX
