# Supplementary material for: Calcium Ionophore-Induced Extracellular Vesicles Mediate Cytoprotection against Simulated Ischemia/Reperfusion Injury in Cardiomyocyte-Derived Cell Lines by Inducing Heme Oxygenase 1
Source: Int J Mol Sci. 2020 Oct 16;21(20):7687. doi: 10.3390/ijms21207687 (PMC7589052; doi:10.3390/ijms21207687)
Supplement: Supplementary file 1 [file ijms-21-07687-s001.pdf]

Supplementary Materials

Calcium Ionophore-Induced Extracellular Vesicles Mediate Cytoprotection against Simulated Ischemia/Reperfusion Injury in Cardiomyocyte-Derived Cell Lines by Inducing Heme Oxygenase 1

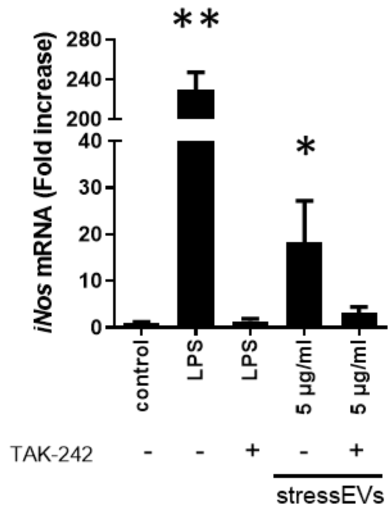

Figure S1. StressEV activate iNOS expression in H9c2 cells. H9c2 cells were stimulated for 6 h with stressEVs (5 or 10 µg/mL) or with LPS (10 ng/mL) in absence or presence of TAK-242 (2.5 µM). *iNos* mRNA levels were determined using qPCR. Data are pooled from three independent experiments ($n = 5$; * $p < 0.05$ vs. control; ** $p < 0.01$ vs. control).
